# Chronic inducible urticaria: confirmation through challenge tests and response to treatment

**DOI:** 10.31744/einstein_journal/2020AO5175

**Published:** 2020-07-03

**Authors:** Amanda Rocha Firmino Pereira, Antônio Abílio Motta, Jorge Kalil, Rosana Câmara Agondi

**Affiliations:** 1 Hospital das Clínicas Faculdade de Medicina Universidade de São Paulo São PauloSP Brazil Hospital das Clínicas, Faculdade de Medicina, Universidade de São Paulo, São Paulo, SP, Brazil.; 2 Faculdade de Medicina Universidade de São Paulo São PauloSP Brazil Faculdade de Medicina, Universidade de São Paulo, São Paulo, SP, Brazil.

**Keywords:** Urticaria, Angioedema, Diagnosis, Drug therapy, Dose-response relationship, drug

## Abstract

**Objective:**

To evaluate the positivity of challenge tests of patients suspected of chronic inducible urticaria and the response to treatment.

**Methods:**

A retrospective study of electronic medical records of patients suspected of chronic inducible urticaria. All patients were submitted to challenge tests with triggering stimuli, according to the clinical history and, subsequently, the response to drug treatment was evaluated.

**Results:**

A total of 191 patients with suspected chronic inducible urticaria were included. It was confirmed in 118 patients and 122 positive tests (4 patients with 2 different positive tests). Most had dermographic urticaria (70.3%), followed by cholinergic urticaria (17.8%). Regarding treatment, 28% responded to antihistamine in licensed doses, 34.7% with increased doses, 9.3% responded to the addition of another medication. The concomitance of chronic inducible urticaria and chronic spontaneous urticaria was found in 35.3% of patients, being more frequent in females, with longer time to control symptoms and higher frequency of cholinergic urticaria.

**Conclusion:**

The confirmation of chronic inducible urticaria in patients with this suspicion, after challenge tests, was high. There was a good response to antihistamine. In the concomitance of chronic spontaneous urticaria, longer time to control symptoms and higher frequency of cholinergic urticaria were observed.

## INTRODUCTION

Urticaria is characterized by the presence of weals, angioedema, or both, and is classified as acute or chronic, according to its duration. Chronic urticaria is characterized by the presence of symptoms, daily or on most days of the week, for more than 6 weeks. chronic urticaria can be classified as spontaneous or inducible, as per the identification of a specific stimulus.^(1)^

Chronic inducible urticaria (CIndU) is characterized by the need for a specific trigger, such as physical stimuli (dermographic urticaria, hot contact urticaria, cold urticaria, delayed pressure urticaria, solar urticaria, and vibratory angioedema) and non-physical stimuli (cholinergic urticaria, contact urticaria, and aquagenic urticaria).^(1,2)^

Chronic inducible urticaria is common, with an estimated prevalence between 0.1 and 0.5% of general population. The most affected age group is between 20 and 40 years.^(3)^ Silpa-archa et al.,^(4)^ observed that 7.2% of patients with chronic urticaria presented with the inducible type, and symptomatic dermographism or dermographic urticaria was the most prevalent among CIndU. The association among the various types of chronic urticaria is common. Sánchez et al.,^(5)^ observed that up to 36% of patients with chronic spontaneous urticaria (CSU) reported concomitant physical triggers.^(5)^

The pathogenesis of CIndU depends on the release of histamine and other active mastocyte mediators. Many studies suggested the participation of autoantigens formed by environmental stimuli, leading to the formation of IgEs that would recognize them.^(3,6)^

The most common CIndU is dermographic, which is characterized by the appearance of linear erythematous and pruritic weals, after friction (scratches or skin friction).^(6,7)^ Delayed pressure urticaria is characterized by the delayed appearance of edema, erythema, and pruritus, a burning sensation, and pain, located in the skin areas exposed to a vertical pressure. Cold urticaria is characterized by the appearance of erythema, weals and pruritus or angioedema when there is exposure to cold air, liquids, or an object.^(8)^

Cholinergic urticaria is a frequent CIndU comprising up to 7% of all inducible urticaria, characterized by the appearance of punctiform erythematous and itchy weals, measuring from 1 to 5mm. Lesions appear with the increase in body temperature, such as with physical exercise, hot baths, and emotional stress.^(3,8)^

Other rarer CIndU types include solar, heat contact, and vibratory angioedema, triggered by solar radiation, contact with heat, and vibration, respectively.^(3,8)^

Chronic urticaria including spontaneous and inducible, has been associated with a negative impact on different aspects of patients’ quality of life.^(9,10)^ Despite the prevalence of chronic urticaria, the evidence on the associated economic and humanistic load in the Brazilian population is still limited. A Brazilian study showed that adults with chronic urticaria present with substantially worse results than do people who live without chronic urticaria relative to quality of life, anxiety, and sleep difficulties. Chronic urticaria, has also been associated with significant losses in work, and with a high-level usage of health resources. The disease has been associated with significantly higher chances of any medical or emergency consultation, or hospitalization.^(11)^ The degree to which the quality of life is hindered varies according to the etiology and severity of the chronic urticaria, and chronic delayed pressure urticaria has affected quality of life more significantly that isolated chronic urticaria.^(12)^

The diagnosis of CIndU is based on the clinical history and challenge tests. The objectives of the challenge tests are to determine the relevant stimulus and evaluate the threshold for that stimulus.^(2)^ Its management involves avoiding the triggering factors, and the symptomatic treatment is the same as recommended for the treatment of CSU, that is, to use the first line of treatment, such as second generation anti-histamines (AH1) at licensed doses. If there is no response, the second line of treatment should be used, increasing the dose of the second generation AH1 up to four times a day. When the patient does not respond to the AH1, omalizumab is the third line of treatment and, after 6 months, when the patient does not respond to this medication, the fourth line of treatment is cyclosporine.^(1)^

## OBJECTIVE

To evaluate the positivity of challenge tests carried out in patients with a presumptive diagnosis of chronic inducible urticaria and response to drug treatment.

## METHODS

This was a retrospective and descriptive study based on electronic patient records, carried out during years 2003 to 2018. Enrolled patients were those with clinical suspicion of CIndU, based on international consensus,^(1,2,13)^ adults (aged >18 years), and of both sexes. Patients with a clinical suspicion of CIndU who had been referred to the Clinical Immunology and Allergy Department of the *Hospital das Clínicas da Faculdade de Medicina da Universidade de São Paulo* (USP), a public tertiary care service, were evaluated considering confirmation of this diagnosis, its progression, and response to treatment.

To confirm diagnosis, the challenge tests were performed in all patients. The demographic data, results of challenge tests, concomitance of CSU, the medications used, medication that led to control of CIndU, and time of treatment for the control of the disease were evaluated. The disease was considered controlled when the patient remained asymptomatic for more than 1 month.

Results of the challenge tests are influenced by various factors, including treatment of patients. Therefore, the symptomatic treatment of the patients was discontinued before the test. The AH1 were interrupted for at least 3 days before, and the corticosteroids, when in use, 7 days before the tests.^(2)^

**Figure 1:**
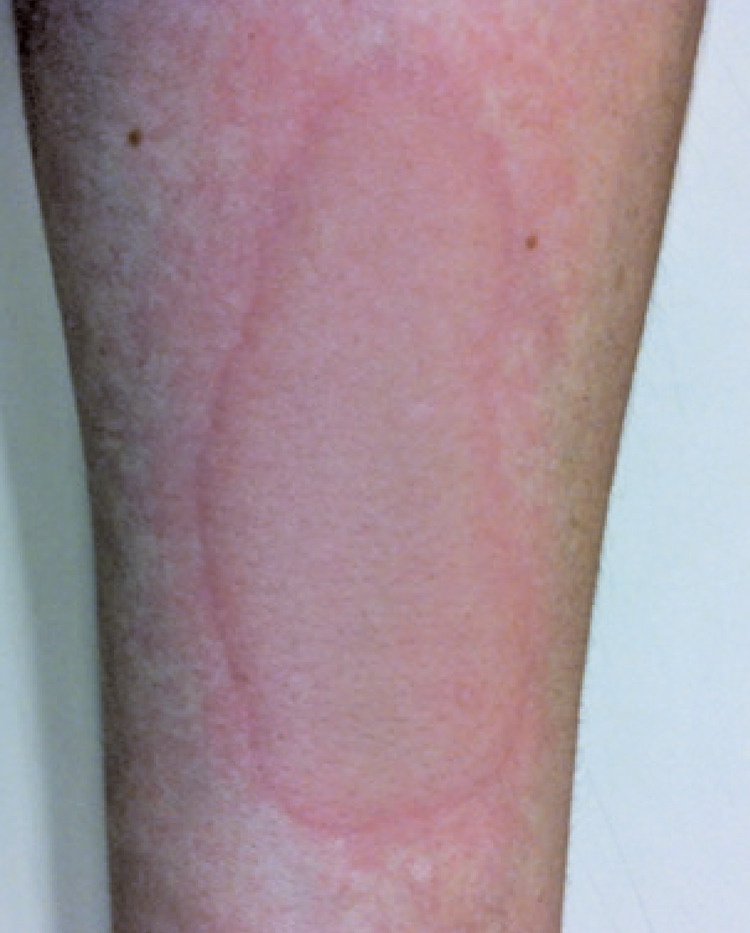
Positive test for cold urticaria, with presence of erythematous and edematous plaque in the volar region of the patient’s forearm

**Figure 2:**
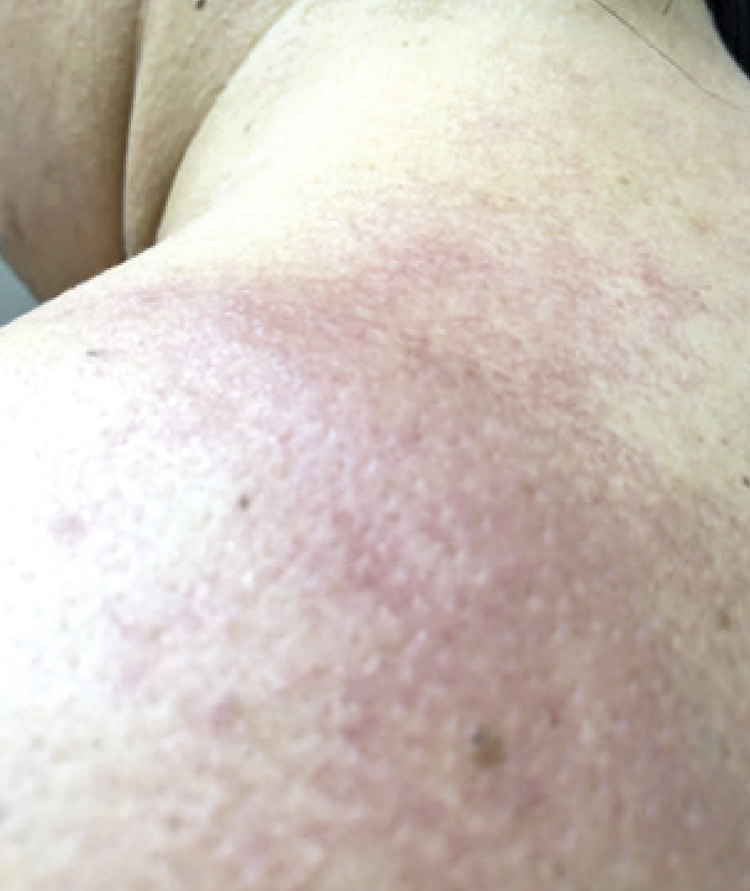
Positive test for delayed pressure urticaria, with the presence of edema and erythema on the patient’s left shoulder

**Figure 3:**
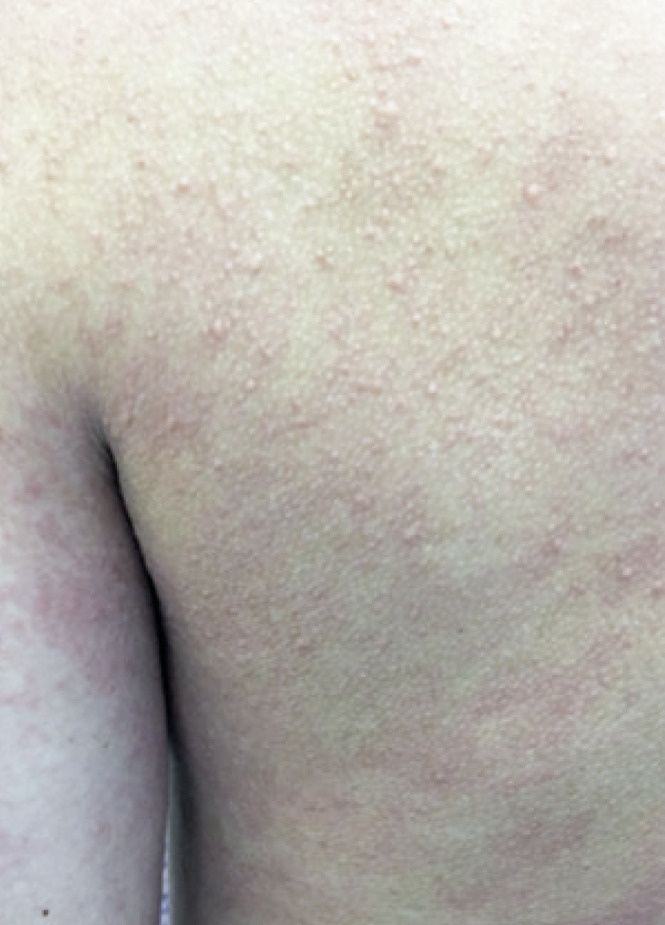
Positive test for cholinergic urticaria, with punctiform erythematous weals on the patient’s dorsum

Patients were submitted to tests according to the clinical history reported. The dermographism test was done on the volar surface of the patients’ forearm, with moderate pressure, using a smooth flat object and/or FricTest^®^, a dermographometer with reading in 10 minutes, which is considered positive in the presence of linear weal and itching.^(1)^ The test for heat contact urticaria was performed by placing the patient’s forearm into a tub of water at 45°C, for 5 minutes, with reading 10 minutes later, and is considered positive in the presence of weal and itching at the site.^(1)^ The test for cold urticaria was carried out by placing an ice cube in a plastic bag on the volar region of the forearm, for 5 minutes, with reading after 10 minutes, and is positive in the presence of weal and itching (Figure 1).^(1)^ For the delayed pressure urticaria test, the Warin test was used by applying 4kg for 5 minutes over the skin of the upper third of the forearm or test with 7kg of pressure divided into 3.5kg each weight, connected by a strip 3cm wide by 15 minutes over the shoulder or thigh, both reading after 6 hours, and considered positive with the presence of local edema and erythema (Figure 2).^(14)^ In the solar urticaria test, a small dorsal area was exposed at 10cm away from the visible light from a slide projector for 10 minutes, with readings at 10, 20, and 30 minutes after the end of the test; it was considered positive if there were weals, erythema, and itching, or a burning sensation.^(1)^ The vibratory test was not performed since there were no patients suspected to have this condition at our service. The test for cholinergic urticaria was carried out by submitting the patient to walk on the treadmill until sweating began, and then 15 minutes of the test were counted out and the reading was done immediately after the test ended and 10 minutes later (Figure 3).^(13)^ Aquagenic urticaria was assessed by applying gauzes wetted with room temperature water on the patient’s dorsum for 20 minutes; it was considered positive in the presence of papules formed in up to 10 minutes.^(15)^

The project was approved by the Research Ethics Committee, CAAE: 79655117.1.0000.0068 and official opinion 2.391.902. Before performing each test, the Informed Consent Form was applied.

For the statistical analysis, all the data analyzed used nonparametric tests. Fisher’s test was used to compare the frequencies of the female sex, response to AH1 (licensed and increased doses), frequency of refractoriness to AH1, and frequency of the type of CIndU among the groups, CIndU, and CIndU associated with CSU. The Mann-Whitney test was used to compare demographic data of the patients, such as current age, age at onset of the disease, and time of the disease. The Kruskal-Wallis test was used to evaluate the clinical comparison between the primary subtypes of inducible urticaria.

## RESULTS

A total of 191 patients was enrolled in this study. Of these, 158 patients (82.7%) were female, mean age of 41.8 years (standard deviation – SD of 13.6 years), mean age at onset of symptoms was 36.1 years (SD of 15.0 years), and mean time of disease of 5.8 years (SD of 7.8 years).

As to the suspected CIndU, 133 patients (69.6%) presented with a hypothesis of symptomatic dermographism, 43 (22.5%) had a suspicion of cholinergic urticaria, 23 (12.0%) to cold, 15 (7.9%) to heat, 15 (7.9%) to delayed pressure, 7 (3.7%) solar, and 5 (2.6%) aquagenic urticaria. Of these patients, 41 (21.5%) were suspected of having two or more associated CIndU. Seventy-three (38.2%) patients presented with a history of concomitant CSU.

All the patients were referred to challenge tests, according to the suspected stimulus. Although instructed about the preparation for the tests, 33 patients (17.3%) were not submitted to the challenge tests due to some contradiction at the time. Moreover, two other patients referred with suspected two or more types of CIndU, were not submitted to at least one of the specific tests.

After referral to challenge tests, CIndU was confirmed in 118 patients (74.7%), and four patients (2.5%) confirmed positivity for two subtypes. The demographic data of patients with CIndU, confirmed by means of challenge tests, are shown on table 1.

**Table 1:** Demographic characteristics of patients with chronic inducible urticaria

Patient characteristics (n=118)*	Results
Female sex	81.4
Current age, years	41.2±13.1
Age at onset, years	36.5±14.7
Time of disease, years	4.9±6.0

Results expressed by % or mean±standard deviation.

^*^Mann-Whitney test.

**Figure 4:**
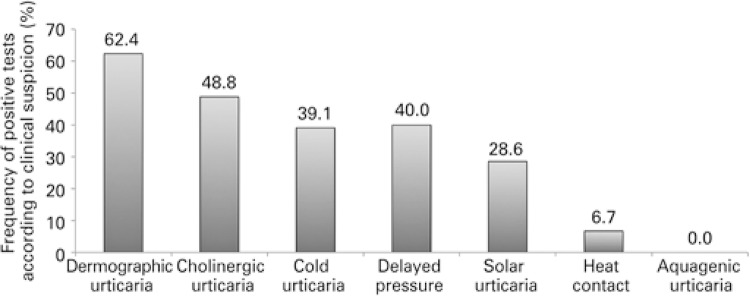
Positivity of challenge tests according to clinical suspicion of chronic inducible urticaria

Diagnosis was confirmed as per the clinical suspicion in 83/133 patients (62.4%) with suspected symptomatic dermographism; 21/43 patients (48.8%) with suspected cholinergic urticaria; 9/23 patients (39.1%) with suspected cold urticaria; 6/15 patients (40.0%) with suspected delayed pressure urticaria, 2/7 patients (28.6%) with suspected solar urticaria, and 1/15 patient (6.7%) confirmed the suspicion of heat contact urticaria. No patient with suspected aquagenic urticaria presented with a positive test, and four patients presented with two concomitant CIndU. Figure 4 shows the frequency of positivity of the challenge tests, as per clinical suspicion.

Of the total 118 patients (and 122 positive tests) with CIndU confirmed by challenge tests, symptomatic dermographism was present in 70.3%; cholinergic urticaria in 17.8%; cold contact in 7.6%; delayed pressure in 5.1%; solar in 1.7%; and heat contact in 0.8%.

As to response to treatment, considering that omalizumab was not available for these patients in our outpatient service of the 118 patients with CIndU confirmed by the challenge tests, most responded to AH1; in that, 32 patients (27.1%) responded to licensed doses of AH1, 41 (34.7%) to increased doses of AH1, and 11 (9.3%) to the addition of another medication to AH1. At the end of this study, 34 patients (28.9%) with no response to AH1 had also not responded to the addition of another medication to AH1.

The primary subtypes of induced urticaria were dermographic, cholinergic, cold, and delayed pressure. The primary clinical differences among them were that dermographic urticaria was the most frequent; in cholinergic urticaria, the male sex was more frequently involved than in other subtypes, as well as a lower response to treatment (AH1 and others) until the time this study was finalized (38%); cold contact urticaria was the one that presented with the shortest history of urticaria, greater frequency of response to AH1 at licensed doses (once a day), and shorter time to control the urticaria; in delayed pressure urticaria, all patients were female, none presented with control of urticaria with the use of AH1 at licensed doses, and time to reach clinical control was longer. These data can be observed on table 2.

**Table 2:** Clinical comparison among the primary subtypes of inducible urticaria

Subtypes of urticaria *versus* clinical characteristics	Dermographic urticaria	Cholinergic urticaria	Cold urticaria	Delayed pressure urticaria	p value
Female sex	85.5	61.9	88.9	100	NS
Current age, years	42.6±12.7	36.9±13.9	37.7±16.7	41.3±11.2	NS
Time of urticaria, years	4.0±4.7	7.0±7.1	2.3±1.5	10.5±13.0	NS
Response to AH1 at a licensed dose	28.9	14.3	44.4	0	0.012
Response to AH1 at a dose 4-fold higher than the licensed dose	36.1	28.6	22.2	66.7	0.012
Refractory to AH1	8.4	19.1	0	16.7	0.012
No response to treatment	26.6	38	33.4	16.6	0.012
Months to response to treatment	17.0±15.8	17.8±17.7	10.2±4.5	19.2±6.6	NS

Kruskal-Wallis Test. Results expressed as % or mean±standard deviation. NS: not significant. AH1: antihistamine.

**Figure 5:**
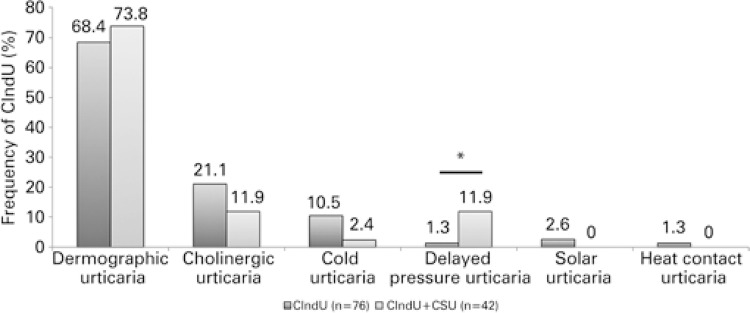
Frequency of chronic inducible urticaria according to concomitance or not of chronic spontaneous urticaria

Concomitance of CIndU and CSU was present in 35.3% of patients. When the patients who only had CIndU were compared to those with a concomitant diagnosis of CSU, the second group presented with a higher frequency of females, longer time needed for symptom control (Table 3, Figure 5), and greater frequency of cholinergic urticaria.

**Table 3:** Comparison between patients with isolated chronic inducible urticaria and those with chronic inducible urticaria associated with chronic spontaneous urticaria

Demographic characteristics	Chronic inducible urticaria (n=76)	Chronic inducible urticaria + chronic spontaneous urticaria (n=42)	p value
Female sex*	73.7	95.2	0.03
Current age, years^†^	41.3±13.5	41.0±12.5	NS
Age at onset, years^†^	36.9±14.7	35.6±14.6	NS
Time of disease, years^†^	4.5±5.2	5.7±7.2	NS
Response to treatment*			
Response to AH1 at licensed dose	32.9	19.0	NS
Response to AH1 at a dose up to 4-fold higher than the licensed dose	30.3	42.9	NS
Refractory to AH1	36.8	38.1	NS
Time to response to treatment, months^†^	15.0	19.8	0.02

* Fisher’s test. ^†^ Mann-Whitney test.

Results expressed by % or mean±standard deviation.

AH1: antihistamine; NS: not significant.

* p=0.02. CIndU: chronic inducible urticaria.

**Figure 6:**
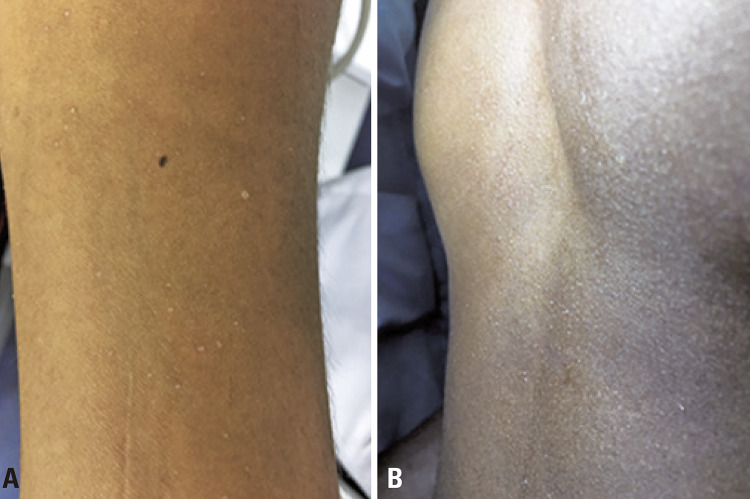
Crystalline miliaria confirmed after challenge tests for cholinergic urticaria. (A) Significant sweating and vesicles with crystalline content on the limbs. (B) Significant sweating and vesicles with crystalline content on the trunk

Two patients with suspected cholinergic urticaria and negative challenge tests presented with significant sudoresis and the presence of microvesicles on the trunk and limbs minutes after the end of the test; the presumptive diagnosis was crystalline miliaria (Figures 6A and 6B).

## DISCUSSION

Chronic inducible urticaria, in contrast with chronic spontaneous, is characterized by the need for specific triggers for the development of weals, angioedema, or both. The signs and symptoms are generally confined to areas exposed to the specific trigger.^(2)^

Our study evaluated 191 patients referred to the service with suspected CIndU. Excluding patients with contraindications (33 patients); all were referred for challenge tests with the trigger suggested by the clinical history. Chronic inducible urticaria was confirmed in 118 patients (74.7%), and 25.3% of patients had their clinical suspicions not confirmed, demonstrating the importance of performing the challenge tests.

Chronic inducible urticaria are diagnosed based on the patient’s history and on challenge test results. It is important to identify and precisely characterize the trigger stimulus and the triggering thresholds of symptoms in these patients, since CIndU can cause severe compromise to quality of life and even have important occupational implications.^(1,2)^

It is important to know that a combination of CIndU and CSU is frequently observed. Curto-Barredo et al.,^(16)^ noted this concomitance in 20% of patients. Our study observed such a concomitance in 35.3% of patients evaluated. These patients were mostly female, took a longer time to control their symptoms, and had a greater frequency of cholinergic urticaria than did the group with isolated CIndU. This study also noted that the occurrence of two or more forms of CIndU, confirmed by challenge tests, was observed in four patients (3.4%).

Data on prevalence, incidence, and duration of CIndU found in literature, are based on observational studies of small populations.^(2)^ Silpa-archa et al.,^(4)^ observed that the frequency of the female sex in patients with physical urticaria was 74.4%. In our sample, we found an even greater frequency in the cases of CIndU confirmed by challenge tests, *i.e.*, of 81.4% of women.

The prevalence of symptomatic dermographism was between 50% and 78% of patients with CIndU, which is consistent with our study that found 70.3%. Cold urticaria was reported in 8% to 37%, and in our study, it was confirmed in 7.6%; for delayed pressure urticaria, from 3% to 20%, and 5.1% in our patients. Cholinergic urticaria has a prevalence of 6% to 13% among CIndU cases, diverging from our study that showed a higher prevalence of 17.8%. Solar urticaria, heat contact, aquagenic, contact, and vibratory urticaria are very rare, and data are limited, as well as those found in our study that observed 1.7% for solar urticaria, 0.8% for heat contact urticaria, and no patient was confirmed as aquagenic urticaria.^(3)^

Our study noted that the patients with CIndU presented with disease duration of 4.9 years, consistent with the literature.^(3)^

Management should be concentrated on avoiding the trigger and on symptomatic treatment, with the objective of reaching complete control of signs and symptoms. To avoid the triggering stimuli is desirable, but, in most cases, it is very difficult to be attained. In addition, for many patients, the threshold for triggering symptoms is low. Detailed information on the properties of the stimulus should allow the patient to recognize and control its expression in normal daily living.^(1,2)^

The second generation AH1 should be considered as first line symptomatic treatment for urticaria, due to their good safety profile. If the symptoms persist with the standard dose, it is recommended to use the same treatment algorithm as for CSU, which is to increase the doses of these AH1 up to four times, as second line of treatment. However, about 50% of patients did not respond to AH1.^(1)^

Studies have demonstrated that patients with CIndU presented with a worse response to AH1 at licensed doses that did those with CSU.^(17-19)^ Kocatürk et al.,^(17)^ observed that 20.9% of patients with CIndU showed symptom control using AH1 at licensed doses, and 37.9% of patients with CSU presented with this response. On the other hand, the rates of response to the quadruplicated dose of AH1 in the two groups were not significantly different between them.

Nonetheless, in our study, 73 patients obtained control with AH1 (61.9%), and of these, 32 patients (27.1%) needed only licensed doses of AH1 to control the disease. Although a larger portion of the patients with concomitant CSU had controlled the clinical picture only with AH1 at doses superior to those licensed, there was no difference as compared to the group with only CIndU.

Studies have shown that omalizumab was effective in the treatment of CIndU refractory to AH1,^(20,21)^ but for our study, this medication was not available. The other medications utilized for patients refractory to AH1 in our study were montelukast, cyclosporine A, and ranitidine. With these medications, control of the disease was achieved in 11 patients (9.3%). The rest of them (34 patients; 28.8%) did not reach control of the disease until the end of this study.

Another therapeutic option would be desensitization, which is indicated only for cold urticaria,^(22,23)^ heat contact urticaria,^(24)^ and solar urticaria.^(25)^ Nonetheless, this induction of tolerance is not long lasting, and daily exposure to the triggering stimulus is necessary. In our study, no patient was submitted to desensitization protocols.

In the national literature, we found no studies on the prevalence of inducible urticaria, in general. There is publication about a Brazilian study conducted by our service about isolated dermographic urticaria related to autoimmune diseases.^(26)^ Additionally, two studies have shown that chronic urticaria seriously compromises the quality of life of patients, due to the debilitating symptoms, which can last for years.^(23,25)^ In one of the studies, 59.8% of patients needed ongoing treatment with AH1.^(10)^

A Brazilian review article mentioned that physical weals are skin conditions resulting from the presence of mastocyte with decreased threshold for degranulation, induced by environmental physical triggering factors, whether located or diffuse, classic or atypical, acquired or familial, with or without the participation of IgE, with variable durations, that can worsen with stress and disappear spontaneously.^(27)^ The clinical pictures of urticaria that, in general, involve complex pathogenesis, clinical progression and complex therapies, can, in some situations, be accompanied by the risk of death; in fact, systemic symptoms can occur during severe episodes. Finally, inducible urticaria has a deep impact on the life of patients, making it important that every healthcare professional have at least a basic knowledge about this disease.^(28)^ A precise diagnosis is needed, in addition to quantification of the clinical expression, establishing specific drug therapy accompanied by a global prevention method, and whenever possible, of physical tolerance induced by desensitization process.^(27)^

One limitation of our study was the impossibility of performing inducible urticaria tests in a large portion of patients (28%). Often, this situation was a consequence of non-collaboration of the patient, but on other occasions, the tests were not done because of the patient’s compromised physical condition, such as, for example, testing for cholinergic urticaria on a treadmill. Another deficiency of our study referred to the availability of medications indicated for treatment of inducible urticaria. This was because, even though supported by medical literature, some were not licensed for use, and other times, it was due to contraindications for their use in our patients.

## CONCLUSION

Confirmation of chronic inducible urticaria in patients with suspicion of the disease is necessary by means of specific and standardized challenge tests, since a large portion of the patients did not show this confirmation. The frequency of response to antihistamine was high. Concomitance of chronic spontaneous urticaria was high and was associated with a longer time to the control of symptoms and to the higher frequency of cholinergic urticaria.
