# Supplementary material for: Chronic inducible urticaria: confirmation through challenge tests and response to treatment
Source: Einstein (Sao Paulo). 2020 Jul 3;18:eAO5175. doi: 10.31744/einstein_journal/2020AO5175 (PMC7334002; doi:10.31744/einstein_journal/2020AO5175)
Supplement: Translation

# Translation: Urticária crônica induzida: confirmação por testes de provocação e resposta ao tratamento

**Authors:** Amanda Rocha Firmino Pereira, Antônio Abílio Motta, Jorge Kalil, Rosana Câmara Agondi

**Affiliations:** 1 Brasil Hospital das Clínicas, Faculdade de Medicina, Universidade de São Paulo, São Paulo, SP, Brasil.; 2 Brasil Faculdade de Medicina, Universidade de São Paulo, São Paulo, SP, Brasil.

**Keywords:** Urticária, Angioedema, Diagnóstico, Tratamento farmacológico, Relação dose-resposta a droga

## Abstract

**Objetivo:**

Avaliar a positividade dos testes de provocação de pacientes com suspeita de urticária crônica induzida e sua resposta ao tratamento.

**Métodos:**

Estudo retrospectivo de prontuários eletrônicos de pacientes com suspeita de urticária crônica induzida. Todos os pacientes foram submetidos aos testes de provocação com estímulos desencadeantes, conforme história clínica e, posteriormente, foi avaliada a resposta ao tratamento medicamentoso.

**Resultados:**

Foram incluídos 191 pacientes com suspeita de urticária crônica induzida, a qual foi confirmada em 118 pacientes e 122 testes positivos (4 pacientes com 2 testes positivos diferentes). A maioria apresentava urticária dermográfica (70,3%), seguida de urticária colinérgica (17,8%). Em relação ao tratamento, 28% responderam ao anti-histamínico em doses licenciadas, 34,7% em doses aumentadas e 9,3% responderam à adição de outro medicamento. A concomitância de urticária crônica induzida com urticária crônica espontânea foi encontrada em 35,3% dos pacientes, sendo mais frequente no sexo feminino, com tempo mais prolongado para controle dos sintomas e maior frequência de urticária colinérgica.

**Conclusão:**

A confirmação de urticária crônica induzida nos pacientes com suspeita da doença foi elevada. Houve boa resposta ao anti-histamínico. Na concomitância com urticária crônica espontânea, observou-se maior tempo para o controle dos sintomas e maior frequência de urticária colinérgica.

## INTRODUÇÃO

A urticária se caracteriza pela presença de urticas, angioedema ou ambos e é classificada em aguda ou crônica, conforme seu tempo de duração. A urticária crônica se caracteriza pela presença de sintomas, diariamente ou na maioria dos dias da semana, por mais de 6 semanas. A urticária crônica pode ser classificada em espontânea ou induzida, conforme a identificação de um estímulo específico.^(1)^

A urticária crônica induzida caracteriza-se pela necessidade de um desencadeante específico como estímulos físicos (urticária dermográfica, de contato ao calor, de contato ao frio, de pressão tardia, solar e angioedema vibratório) e não físicos (urticária colinérgica, de contato e aquagênica).^(1,2)^

A urticária crônica induzida é comum, com prevalência estimada entre 0,1 a 0,5% da população geral. A faixa etária mais comprometida está entre 20 e 40 anos.^(3)^ Silpa-archa et al.,^(4)^ observaram que 7,2% dos pacientes com urticária crônica apresentavam o tipo induzido, e o dermografismo sintomático ou urticária dermográfica era a mais prevalente dentre a urticária crônica induzidas. Não é incomum a associação entre os vários tipos de urticária crônica. Sánchez et al.,^(5)^ observaram que até 36% dos pacientes com urticária crônica espontânea relatavam desencadeantes físicos concomitantes.^(5)^

A patogênese das urticária crônica induzidas depende da liberação de histamina e outros mediadores de mastócitos ativados. Muitos estudos sugerem participação de autoantígenos formados por estímulos ambientais levando a formação de IgEs que os reconheceria.^(3,6)^

A urticária crônica induzida mais comum é a dermográfica, que se caracteriza pelo aparecimento de urticas lineares eritematosas e pruriginosas, após fricção (arranhando ou atritando a pele).^(6,7)^ A urticária de pressão tardia caracteriza-se pelo aparecimento de edema, eritema e prurido, queimação e dor, localizados nas áreas de pele expostas a uma pressão vertical. A urticária ao frio se caracteriza pelo aparecimento de eritema, urtica e prurido ou angioedema quando exposição ao ar, a líquidos ou a um objeto frio.^(8)^

A urticária colinérgica é uma urticária crônica induzida frequente, compreende até 7% de todas as urticárias induzidas, caracteriza-se pelo aparecimento de urticas eritematosas puntiformes com 1 a 5mm e pruriginosas. As lesões surgem com o aumento da temperatura corporal, como exercício físico, banho quente e estresse emocional.^(3,8)^

Outras urticária crônica induzidas mais raras incluem a solar, ao calor e o angioedema vibratório, desencadeadas pela radiação solar, contato ao calor e vibração, respectivamente.^(3,8)^

A urticária crônica, incluindo a espontânea e a induzida, tem sido associada a um impacto negativo em diferentes aspectos da qualidade de vida dos pacientes.^(9,10)^ Apesar da prevalência de urticária crônica, as evidências sobre a carga econômica e humanística associada na população brasileira ainda são limitadas. Estudo brasileiro mostrou que adultos com urticária crônica apresentam resultados substancialmente piores do que pessoas que vivem sem urticária crônica com relação a qualidade de vida, ansiedade e dificuldades para dormir. A urticária crônica também foi associada a prejuízos significativos nas atividades laborais, e ao uso de recursos de saúde bastante elevado. A doença tem sido associada a chances significativamente maiores de qualquer consulta médica, consulta de emergência ou hospitalização.^(11)^ O grau em que a qualidade de vida é afetada varia de acordo com a etiologia e a gravidade da urticária crônica, e a urticária crônica associada à urticária de pressão tardia afetou a qualidade de vida mais significativamente do que a urticária crônica isolada.^(12)^

O diagnóstico de urticária crônica induzida se baseia na história clínica e em testes de provocação. Os objetivos dos testes de provocação são determinar o estímulo relevante e avaliar o limiar para este estímulo.^(2)^ O manejo envolve evitar os desencadeantes, e o tratamento sintomático é o mesmo recomendado para o tratamento da urticária crônica espontânea, ou seja, usar a primeira linha de tratamento, com anti-histamínico (AH1) de segunda geração em doses licenciadas. Não havendo resposta, deve-se usar a segunda linha de tratamento, aumentando a dose do AH1 de segunda geração até quatro vezes ao dia. Quando o paciente não responde ao AH1, o omalizumabe é a terceira linha de tratamento e, após 6 meses, quando o paciente não responde a este medicamento, a quarta linha de tratamento é a ciclosporina.^(1)^

## OBJETIVO

Avaliar a positividade dos testes de provocação realizados em pacientes com hipótese diagnóstica de urticária crônica induzida e a resposta ao tratamento medicamentoso.

## MÉTODOS

Estudo retrospectivo, descritivo e baseado em prontuário eletrônico, realizado durante os anos de 2003 a 2018. Foram incluídos pacientes com suspeita clínica de urticária crônica induzida, baseado nos consensos internacionais,^(1,2,13)^ adultos (>18 anos de idade) e de ambos os sexos. Avaliaram-se pacientes com suspeita clínica de urticária crônica induzida encaminhados ao Serviço de Imunologia Clínica e Alergia do Hospital das Clínicas da Faculdade de Medicina da Universidade de São Paulo (USP), um serviço público terciário, quanto à confirmação deste diagnóstico, sua evolução e resposta ao tratamento.

Para a confirmação diagnóstica, os testes de provocação foram realizados em todos os pacientes. Os dados demográficos, o resultado destes testes de provocação, a concomitância de urticária crônica espontânea, os medicamentos utilizados, o medicamento que levou ao controle da urticária crônica induzida e o tempo de tratamento para o controle da doença foram avaliados. A doença foi considerada controlada quando o paciente permanecia assintomático por mais de 1 mês.

Os resultados dos testes de provocação são influenciados por vários fatores, incluindo o tratamento dos pacientes. Assim, o tratamento sintomático dos pacientes foi descontinuado antes do teste. Os AH1 foram interrompidos pelo menos 3 dias antes do teste e os corticosteroides, quando em uso, 7 dias antes do teste.^(2)^

Os pacientes foram submetidos a testes conforme a história clínica relatada. O teste do dermografismo foi realizado na face volar do antebraço dos pacientes, realizando pressão moderada, utilizando-se objeto liso contundente e/ou FricTest^®^, um dermografômetro, com leitura em 10 minutos, sendo considerado positivo se presença de urtica linear e prurido.^(1)^ O teste para urticária de contato ao calor foi realizado colocando-se o antebraço do paciente em uma cuba com água a 45°C, por 5 minutos, com leitura após 10 minutos do término, sendo considerado positivo se presença de urtica e prurido no local.^(1)^ O teste para urticária de contato ao frio foi realizado colocando-se um cubo de gelo protegido em saco plástico na região volar do antebraço, por 5 minutos, com leitura após 10 minutos do término, sendo considerado positivo se presença de urtica e prurido (Figura 1).^(1)^ Para o teste de urticária de pressão tardia, foi utilizado o teste de Warin, aplicando-se pressão de 4kg por 5 minutos sobre na pele do terço superior externo do antebraço ou teste com 7kg de pressão divididos em 3,5kg cada peso, ligados por uma faixa de 3cm de largura por 15 minutos sobre o ombro ou coxa, ambos com leitura após 6 horas, sendo positivo se presença de edema e eritema local (Figura 2).^(14)^ No teste de urticária solar, pequena área do dorso foi exposta a 10cm da luz visível de um projetor de slides por 10 minutos, sendo realizada leituras 10, 20 e 30 minutos após o fim do teste, e sendo considerado positivo se urticas, eritema e prurido, ou queimação.^(1)^ O teste vibratório não foi realizado por não se apresentarem pacientes com a suspeita em nosso serviço. O teste de urticária colinérgica foi realizado submetendo-se o paciente a caminhada na esteira, até que se iniciasse sudorese e fossem, então, contados 15 minutos de teste, sendo a leitura realizada imediatamente e após 10 minutos do término do teste (Figura 3).^(13)^ A urticária aquagênica foi avaliada aplicando-se gazes umedecidas com água em temperatura ambiente no dorso do paciente por 20 minutos, sendo considerado positivo se formação de pápula em até 10 minutos.^(15)^

**Figura 1:** Teste positivo para urticária de contato ao frio, com presença de placa eritematosa e edematosa em região volar do antebraço do paciente

**Figura 2:** Teste positivo para urticária de pressão tardia, com presença de edema e eritema no ombro esquerdo do paciente

**Figura 3:** Teste positivo de urticária colinérgica, com presença de urticas eritematosas puntiforme no dorso do paciente

O trabalho foi aprovado pelo Comitê de Ética em Pesquisa com CAAE: 79655117.1.0000.0068 e número do parecer 2.391.902. Antes da realização de cada teste, foi aplicado Termo de Consentimento Livre e Esclarecido.

Para a análise estatística, todos os dados analisados utilizaram testes não paramétricos. O teste de Fisher foi utilizado para comparar as frequências de sexo feminino, resposta ao AH1 (dose licenciada e aumentada), frequência de refratariedade ao AH1 e frequência de tipo de urticária crônica induzida entre os grupos, urticária crônica induzida e urticária crônica induzida associada a urticária crônica espontânea (UCE). O teste de Mann-Whitney foi utilizado para comparar dados demográficos dos pacientes, como idade atual, idade de início da doença e tempo de doença. O teste de Kruskal-Wallis foi utilizado para avaliar a comparação clínica entre os principais subtipos de urticária induzida.

## RESULTADOS

Um total de 191 pacientes foi incluído neste estudo. Destes, 158 pacientes (82,7%) eram do sexo feminino, com média de idade de 41,8 anos (desvio padrão – DP de 13,6 anos), média de idade de início dos sintomas de 36,1 anos (DP de 15,0 anos) e média de tempo de doença de 5,8 anos (DP de 7,8 anos).

Em relação à suspeita de urticária crônica induzida, 133 pacientes (69,6%) apresentavam hipótese de dermografismo sintomático, 43 (22,5%) tinham suspeita de urticária colinérgica, 23 (12,0%) ao frio, 15 (7,9%) ao calor, 15 (7,9%) de pressão tardia, 7 (3,7%) solar e 5 (2,6%) aquagênica. Destes pacientes, 41 (21,5%), apresentavam suspeita diagnóstica de duas ou mais urticária crônica induzidas associadas. Apresentavam história de urticária crônica espontânea concomitante 73 pacientes (38,2%).

Todos os pacientes foram encaminhados para os testes de provocação, conforme o estímulo suspeito. Embora orientados quanto ao preparo para os testes, 33 pacientes (17,3%) não foram submetidos aos testes de provocação, devido a alguma contraindicação no momento. Além destes, outros dois pacientes encaminhados com suspeita de duas ou mais urticária crônica induzidas não realizaram pelo menos um dos testes específicos.

Após o encaminhamento para os testes de provocação, a urticária crônica induzida foi confirmada em 118 pacientes (74,7%), e quatro pacientes (2,5%) confirmaram a positividade para dois subtipos. Os dados demográficos dos pacientes com urticária crônica induzida confirmada por meio dos testes de provocação estão demonstrados na tabela 1.

**Tabela 1:** Características demográficas dos pacientes com urticária crônica induzida

Características dos pacientes (n=118)*	Resultados
Sexo feminino	81,4
Idade atual, anos	41,2±13,1
Idade de início, anos	36,5±14,7
Tempo de doença, anos	4,9±6,0

Resultados expressos por % ou média±desvio padrão.

^*^ Teste de Mann-Whitney.

O diagnóstico foi confirmado conforme a suspeita clínica em: 83/133 pacientes (62,4%) com suspeita de dermografismo sintomático; 21/43 pacientes (48,8%) com a suspeita de urticária colinérgica; 9/23 pacientes (39,1%) com suspeita de urticária ao frio; 6/15 pacientes (40,0%) com suspeita de urticária de pressão tardia, 2/7 pacientes (28,6%) com suspeita de urticária solar e 1/15 paciente (6,7%) confirmou a suspeita de urticária ao calor. Nenhum paciente com suspeita de urticária aquagênica apresentou teste positivo, e quatro pacientes apresentavam 2 urticária crônica induzidas concomitantes. A figura 4 mostra a frequência de positividade dos testes de provocação, conforme suspeita clínica.

**Figura 4:** Positividade dos testes de provocação conforme suspeita clínica de urticária crônica induzida

Do total de 118 pacientes (e 122 testes positivos) com urticária crônica induzida confirmada por testes de provocação, o dermografismo sintomático estava presente em 70,3%; urticária colinérgica em 17,8%; ao frio em 7,6%; de pressão tardia em 5,1%; solar em 1,7%; e ao calor em 0,8%.

Em relação à resposta ao tratamento, sendo que omalizumabe não estava disponível para estes pacientes, dos 118 pacientes com urticária crônica induzida confirmados pelos testes de provocação, a maioria respondeu aos AH1, sendo que 32 pacientes (27,1%) responderam a doses licenciadas de AH1, 41 (34,7%) a doses aumentadas de AH1 e 11 (9,3%) à adição de outro medicamento ao AH1. Não tinham respondido à adição de outro medicamento ao AH1 até o fim deste estudo 34 pacientes (28,9%) todos sem resposta ao AH1.

Os principais subtipos de urticárias induzidas foram dermográfica, colinérgica, ao frio e de pressão tardia. As principais diferenças clínicas entre elas foram: a urticária dermográfica foi a mais frequente; na urticária colinérgica, o sexo masculino estava mais frequente que nos outros subtipos e foi observada menor resposta ao tratamento (AH1 e outros) até a época de finalização deste estudo (38%); a urticária ao frio foi a que apresentou menor tempo de história de urticária, maior frequência de resposta ao AH1 em doses licenciadas (uma vez ao dia) e menor tempo para o controle da urticária; e, na urticária de pressão tardia, todos os pacientes eram do sexo feminino, nenhum apresentou controle da urticária com o uso de AH1 em doses licenciadas e foi maior o tempo para alcançar o controle clínico. Estes dados podem ser observados na tabela 2.

**Tabela 2:** Comparação clínica entre os principais subtipos de urticária induzida

Subtipos de urticária *versus* características clínicas	Urticária dermográfica	Urticária colinérgica	Urticária ao frio	Urticária de pressão tardia	Valor de p
Sexo feminino	85,5	61,9	88,9	100	NS
Idade atual, anos	42,6±12,7	36,9±13,9	37,7±16,7	41,3±11,2	NS
Tempo de urticária, anos	4,0±4,7	7,0±7,1	2,3±1,5	10,5±13,0	NS
Resposta ao AH1 em dose licenciada	28,9	14,3	44,4	0	0,012
Resposta ao AH1 em dose até 4 vezes maior que a licenciada	36,1	28,6	22,2	66,7	0,012
Refratários ao AH1	8,4	19,1	0	16,7	0,012
Sem resposta ao tratamento	26,6	38	33,4	16,6	0,012
Meses para resposta ao tratamento	17,0±15,8	17,8±17,7	10,2±4,5	19,2±6,6	NS

Teste de Kruskal-Wallis. Resultados expressos por % ou média±desvio padrão. NS: não significativo; AH1: anti-histamínico.

A concomitância de urticária crônica induzida com urticária crônica espontânea esteve presente em 35,3% dos pacientes. Quando os pacientes com apenas urticária crônica induzida foram comparados aos que apresentavam diagnóstico de urticária crônica espontânea concomitante, o segundo grupo apresentava maior frequência de sexo feminino, maior tempo necessário para controle dos sintomas (Tabela 3, Figura 5) e maior frequência de urticária colinérgica.

**Tabela 3:** Comparação entre os pacientes com urticária crônica induzida isolada e aqueles com urticária crônica induzida associada à urticária crônica espontânea

Características demográficas	Urticária crônica induzida (n=76)	Urticária crônica induzida + urticária crônica espontânea (n=42)	Valor de p
Sexo feminino*	73,7	95,2	0,03
Idade atual, anos^†^	41,3±13,5	41,0±12,5	NS
Idade de início, anos^†^	36,9±14,7	35,6±14,6	NS
Tempo de doença, anos^†^	4,5±5,2	5,7±7,2	NS
Resposta ao tratamento*			
Resposta ao AH1 em dose licenciada	32,9	19,0	NS
Resposta ao AH1 em dose até 4 vezes maior que a licenciada	30,3	42,9	NS
Refratários ao AH1	36,8	38,1	NS
Tempo para resposta ao tratamento, meses^†^	15,0	19,8	0,02

* Teste de Fisher; ^†^ Teste de Mann-Whitney.

Resultados expressos por % ou média±desvio padrão.

AH1: anti-histamínico; NS: não significativo.

**Figura 5:** Frequência de urticária crônica induzida conforme a concomitância ou não com urticária crônica espontânea

* p=0,02. UCInd: urticária crônica induzida.

Dois pacientes com suspeita de urticária colinérgica e testes de provocação negativos apresentaram sudorese importante e presença de microvesículas em tronco e membros minutos após o término do teste, e a hipótese diagnóstica foi de miliária cristalina (Figuras 6A e 6B).

**Figura 6:** Miliária cristalina confirmada após testes de provocação para urticária colinérgica. (A) Sudorese importante e vesículas com conteúdo cristalino em membro. (B) Sudorese importante e vesículas com conteúdo cristalino em tronco

## DISCUSSÃO

A urticária crônica induzida, em contraste com a espontânea, é caracterizada pela necessidade de desencadeadores específicos para o desenvolvimento das urticas, do angioedema ou de ambos. Os sinais e sintomas são geralmente confinados a áreas da pele expostas ao gatilho específico.^(2)^

Nosso estudo avaliou 191 pacientes encaminhados ao serviço com suspeita de urticária crônica induzida. Excluindo-se os pacientes com contraindicação (33 pacientes), todos foram encaminhados para testes de provocação com o desencadeante sugerido pela história clínica. A urticária crônica induzida foi confirmada em 118 pacientes (74,7%), e 25,3% dos pacientes não confirmaram suas suspeitas clínicas, demonstrando a importância da realização dos testes de provocação.

As urticária crônica induzidas são diagnosticadas com base no histórico do paciente e nos resultados do teste de provocação. É importante identificar e caracterizar com precisão o estímulo desencadeante e os limiares de desencadeamento dos sintomas destes pacientes, pois as urticária crônica induzidas podem causar grave comprometimento da qualidade de vida e até importantes implicações ocupacionais.^(1,2)^

É importante reconhecer que uma combinação de urticária crônica induzida e espontânea é frequentemente observada. Curto-Barredo et al.,^(16)^ observaram esta concomitância em 20% dos pacientes. Nosso estudo observou tal concomitância 35,3% dos pacientes avaliados. Estes pacientes eram em sua maioria do sexo feminino, tinham tempo mais prolongado para controle dos sintomas e maior frequência de urticária colinérgica do que o grupo com apenas urticária crônica induzida. Este estudo também observou que a ocorrência de duas ou mais formas de urticária crônica induzidas, confirmadas com testes de provocação, foi observada em quatro pacientes (3,4%).

Os dados sobre prevalência, incidência e duração das urticária crônica induzidas, encontrados na literatura, são baseados em estudos observacionais de pequenas populações.^(2)^ Silpa-archa et al.,^(4)^ observaram que a frequência do sexo feminino nos pacientes com urticárias físicas foi de 74,4%. Em nossa amostra, encontramos frequência ainda maior nas urticária crônica induzidas confirmadas com testes de provocação, de 81,4% de mulheres.

A prevalência de dermografismo sintomático encontra-se entre 50% a 78% dos pacientes com urticária crônica induzida, sendo compatível com nosso estudo, que encontrou 70,3%. A urticária ao frio foi relatada em 8% a 37% e, em nosso estudo, foi comprovada em 7,6%; para urticária de pressão tardia, de 3% a 20%, sendo de 5,1% em nossos pacientes. A urticária colinérgica tem prevalência de 6% a 13% entre as urticária crônica induzidas, divergindo de nosso estudo, que mostrou prevalência mais elevada, de 17,8%. As urticária solar, ao calor, aquagênica, de contato e vibratória são muito raras, e os dados são limitados, assim como os encontrados em nosso estudo, que observou 1,7% para urticária solar, 0,8% para urticária ao calor e nenhum paciente apresentou confirmação da suspeita de urticária aquagênica.^(3)^

Nosso estudo observou que os pacientes com urticária crônica induzida apresentavam duração da doença de 4,9 anos, compatível com a literatura.^(3)^

O tratamento deve se concentrar em evitar o desencadeante e no tratamento sintomático, com o objetivo de alcançar o controle completo dos sinais e sintomas. Evitar os estímulos desencadeantes é desejável, mas, na maioria das vezes, é muito difícil de ser alcançado. Ainda, para muitos pacientes, o limiar para desencadear os sintomas é baixo. Informações detalhadas sobre as propriedades do estímulo devem permitir ao paciente reconhecer e controlar a exposição na vida diária normal.^(1,2)^

Os AH1s de segunda geração devem ser considerados como tratamento sintomático de primeira linha para a urticária, devido ao seu bom perfil de segurança. Caso os sintomas persistam com a dosagem padrão, recomenda-se o uso do mesmo algoritmo de tratamento para urticária crônica espontânea, que é aumentar a dose desses AH1 até quatro vezes, como tratamento de segunda linha. Entretanto, cerca de 50% dos pacientes não responderão aos AH1.^(1)^

Estudos demonstraram que pacientes com urticária crônica induzida apresentavam pior resposta aos AH1 em doses licenciadas do que os com urticária crônica espontânea.^(17-19)^ Kocatürk et al.,^(17)^ observaram que 20,9% dos pacientes com urticária crônica induzida apresentaram controle dos sintomas usando AH1 em doses licenciadas, e 37,9% dos pacientes com urticária crônica espontânea apresentaram esta resposta. Já as taxas de resposta à dose de AH1 quadruplicada nos dois grupos não eram significativamente diferentes entre si.

Entretanto, em nosso estudo, 73 pacientes obtiveram controle com AH1 (61,9%), e, destes, 32 pacientes (27,1%), para o controle da doença, necessitaram apenas de doses licenciadas de AH1. Embora parcela maior de pacientes com urticária crônica espontânea concomitante tenha controlado o quadro apenas com AH1 em doses superiores à licenciada, não houve diferença em relação ao grupo com apenas urticária crônica induzida.

Estudos mostraram que o omalizumabe era eficaz no tratamento da urticária crônica induzida refratária ao AH1,^(20,21)^ porém, para nosso estudo, esse medicamento não estava disponível. Os outros medicamentos utilizados para os pacientes refratários ao AH1, em nosso estudo foram montelucaste, ciclosporina A e ranitidina. Com estes medicamentos, o controle da doença foi obtido em mais 11 pacientes (9,3%). O restante deles (34 pacientes; 28,8%) não tinha atingido o controle da doença até o término deste estudo.

Outra opção terapêutica seria a dessensibilização, que está indicada apenas para urticária ao frio,^(22,23)^ ao calor^(24)^ e solar.^(25)^ No entanto, esta indução de tolerância não é duradoura, sendo necessária exposição diária ao estímulo desencadeante. Em nosso estudo, nenhum paciente foi submetido a protocolos de dessensibilização.

Não encontramos, na literatura nacional, estudos sobre prevalência de urticárias induzidas em geral. Há publicação de um estudo brasileiro pelo nosso serviço sobre urticária dermográfica isoladamente com relação a doenças autoimunes.^(26)^ Além disso, dois estudos mostraram que a urticária crônica compromete seriamente a qualidade de vida dos pacientes, devido aos sintomas debilitantes, que podem durar anos.^(23,25)^ Em um dos estudos, 59,8% dos pacientes necessitaram tratamento contínuo com AH1.^(10)^

Artigo de revisão brasileiro cita que as urticárias físicas são dermatoses decorrentes da presença de disfunção mastocitária, associada à diminuição do limiar de desgranulação citoplasmática dos mediadores de anafilaxia, induzidas por fatores físicos ambientais estimulatórios, localizadas ou difusas, clássicas ou atípicas, adquiridas ou familiares, com ou sem a participação da IgE, de duração variável, podendo piorar com o estresse e desaparecer espontaneamente.^(27)^ Os quadros de urticária que, em geral, envolvem patogênese, evolução clínica e terapêutica complexas, podem, em algumas situações, acompanhar-se de risco de morte; de fato, sintomas sistêmicos podem ocorrer durante episódios graves. Finalmente, as urticárias induzidas têm profundo impacto sobre a vida dos pacientes, de forma que é importante que todo o profissional da saúde tenha pelo menos um conhecimento básico sobre essas doenças.^(28)^ Faz-se necessário um diagnóstico preciso, além de ser preciso quantificar a expressão clínica, estabelecer a farmacoterapia específica acompanhada de um plano preventivo global e, quando possível, de tolerância física induzida por meio do processo de dessensibilização.^(27)^

Uma limitação do nosso estudo foi a impossibilidade de realização dos testes de urticária induzida em uma parcela grande dos pacientes (28%). Muitas vezes, esta situação foi devido à não colaboração do paciente, porém, em outras ocasiões, a não realização dos testes ocorreu pela falta de condição física pelo paciente, como, por exemplo, para realização de teste para urticária colinérgica em esteira. Outra deficiência de nosso estudo foi referente à disponibilidade de medicamentos indicados para tratamento de urticária crônica induzida. Isto ocorreu porque alguns, mesmo respaldado pela literatura médica, não eram licenciados para seu uso e, outras vezes, devido à contraindicação para seu uso por nossos pacientes.

## CONCLUSÃO

A confirmação de urticária crônica induzida nos pacientes com suspeita da doença se faz necessária por testes de provocação específicos e padronizados, pois parcela grande de pacientes não apresentou esta confirmação. A frequência de resposta ao anti-histamínico foi alta. A concomitância de urticária crônica espontânea foi elevada e esteve associada a um tempo mais prolongado para o controle dos sintomas e à maior frequência de urticária colinérgica.
